# Factors associated with the unwillingness of Jordanians, Palestinians and Syrians to be vaccinated against COVID-19

**DOI:** 10.1371/journal.pntd.0009957

**Published:** 2021-12-09

**Authors:** Sima Zein, Sarah B. Abdallah, Ahmed Al-Smadi, Omar Gammoh, Wajdy J. Al-Awaida, Hanan J. Al-Zein

**Affiliations:** 1 Faculty of Science, Department of Biology and Biotechnology, American University of Madaba, Madaba, Jordan; 2 Princess Salma Faculty of Nursing, Department of Adult Health Nursing, Al al-Bayt University, Irbid, Jordan; 3 Faculty of Health Sciences, Department of Pharmacy, American University of Madaba, Madaba, Jordan; 4 Faculty of Nursing, Al-Ahliyya Amman University, Amman, Jordan; Colorado State University College of Veterinary Medicine and Biomedical Sciences, UNITED STATES

## Abstract

**Background:**

The COVID-19 pandemic is expected to continue to inflect immense burdens of morbidity and mortality, not to mention the sever disruption of societies and economies worldwide. One of the major challenges to managing COVID-19 pandemic is the negative attitudes towards vaccines and the uncertainty or unwillingness to receive vaccinations. We evaluated the predictors and factors behind the negative attitudes towards COVID-19 vaccines in 3 countries in the Middle East.

**Methods:**

A cross-sectional, self-administered survey was conducted between the 1^st^ and the 25^th^ of December, 2020. Representative sample of 8619 adults residing in Jordan, West Bank, and Syria, completed the survey via the Web or via telephone interview. The survey intended to assess intent to be vaccinated against COVID-19 and to identify predictors of and reasons among participants unwilling/hesitant to get vaccinated.

**Results:**

The total of the 8619 participants included in this study were the ones who answered the question on the intent to be vaccinated. Overall, 32.2% of participants (n = 2772) intended to be vaccinated, 41.6% (n = 3589) didn’t intend to get vaccinated, and 26.2% (n = 2258) were not sure. The main factors associated with the willingness to take the vaccine (yes responses) included females, 18–35 years old, Syrians and Jordanians, a large family size, and having received a flu vaccine last year. Reasons for vaccine hesitancy included the lack of rigorous evaluation of the vaccine by the FDA and the possible long-term health risks associated with the vaccines (the wait-and-see approach).

**Conclusion:**

This survey, conducted in December when the number of cases and deaths per day due to COVID-19 were at or near peak levels of the initial surge in the three regions under investigation. The survey revealed that most of survey’s participants (67.8%) were unwilling/hesitant to get vaccinated against COVID-19 with the lack of trust in the approval process of the vaccine being the main concern; the two main characteristics of those participants were more than 35 years old and participants holding a Bachelor’s degree or higher. Targeted and multi-pronged efforts will be needed to increase acceptance of COVID-19 vaccine in Jordan, West Bank and Syria.

## Author summary

This study investigated vaccine willingness, unwillingness, or hesitancy by assessing the intention to get vaccinated against COVID-19. We found that a considerable percentage of participants are unwilling/hesitant to receive the COVID-19 vaccine. Perceived barriers to vaccination, namely the lack of rigorous evaluation of the vaccines and the possible long-term health risks associated with it, were significant predictors of COVID-19 vaccination intent. A substantial proportion of participants willing to get vaccinated were the 18–35 years old and the participants with a high school diploma or less.

## Introduction

The COVID-19 disease caused by the severe acute respiratory syndrome coronavirus 2 (SARS-CoV-2) is continuing to become a major and rapid global threat, in particular with the emergence of the different variants. As of January, 24th, 2021, the pandemic is affecting more than 218 countries and territories, and approximately 98.9 million people and leading to nearly 2.1 million deaths worldwide [1]. The pandemic has unprecedented strain on healthcare resources, undermined economic activity worldwide, and instilled fear into the general population [2,3]. The pandemic will depend on acquiring immunity in approximately 67% of the population (herd immunity) through natural means, or by allowing a large proportion of the population to become infected, increasing the strain on the healthcare system and the economy [4]. Hence, a predominant vaccination is essential for managing the pandemic, although it is not yet clear what would be the degree and the duration of protection the available vaccines will offer [5]. However, the current pandemic is occurring among a widespread mistrust in the safety and effectiveness of vaccines globally [6], thousands of people have protested around the world against mass vaccination [7].

Vaccine hesitancy and disinformation in many countries are major challenges to achieving coverage and population immunity [5,6]. Anti-vaccination groups in Jordan, West Bank, and Syria, among several other nations, are now lobbying against the vaccines, so governments and public health agencies should be prepared to increase vaccine awareness so that the majorities will get vaccinated in those three regions. We assessed intent to be vaccinated against COVID-19 among a representative sample of adults among Jordanians, Palestinians, and Syrians located in 3 regions sharing the same borders, culture, traditions, beliefs, however, the political and economical instability in Syria and Palestine which caused a major decline in the health system might have affected the peoples’ attitudes toward the vaccine. In addition, we have identified the predictors of intent to decline or hesitancy to get vaccinated and the possible reasons behind it.

## Methods

### Ethics statement

The study has been approved by the Institutional Research Board/Ethical Committee at the American University of Madaba approval number (H20007) by a formal written consent. The study objectives and methods were fully explained to the participants.

### Participants and survey administration

We surveyed a nationally representative sample of adults residing in Jordan, West Bank, and Syria via a survey prepared in Google form in English translated to the local Arabic language which was then circulated via the web or through a phone interview. The Data was analyzed using IBM SPSS version 23.0. Informed consent was obtained at the beginning of the survey. The survey data was collected between the 1st till the 25^th^ of December, 2020.

### Measures

We assessed intent to be vaccinated for the SARS-CoV-2 with the question, “Would you get vaccinated for coronavirus once it gets available in your country?” followed by the response options ‘yes’, ‘no’, and ‘not sure’. Participants who responded ‘no’ or ‘not sure’ were asked to choose one or more of the following reasons for their answer: “I don’t believe in vaccines”; “I need additional information since vaccine wasn’t rigorously evaluated by FDA”; “I will wait till other people try it first”; “I don’t think the coronavirus is a threat”; “I always get side effects when I get vaccinated”; and “Other reasons”.

Data on participant characteristics were provided by participants and included age, sex, nationality and geographic location, educational level, household size, marital status, employment status, receipt of influenza vaccination in the prior year, and the intention of getting the influenza vaccine this year.

### Statistical analysis

Frequencies were calculated to summarize participants’ characteristics and percentages. Cross- tabulations and y^2^ tests were used to estimate unadjusted associations of participant characteristics with the 3 possible outcomes of intent to get vaccinated. To better distinguish characteristics associated with responses of ‘not sure’ versus ‘yes’ and characteristics associated with responses of ‘no’ versus ‘yes’, we also calculated separate y^2^ tests and associated *P* values for these 2 sets of comparisons, with a p-value ≤ 0.05 is statistically significant.

To estimate corresponding adjusted (univariate) associations, we used univariate logistic regression between the acceptances of vaccine versus the refusal (no) or the hesitation (not sure) to identify the influencing factors, with the odds ratio (RRR), and a 95% confidence interval (CI) being calculated.

## Results

Survey respondents represented a random sample of the populations of 3 Middle Eastern countries; Jordan, West Bank and Syria. Most participants (87.2%) completed the survey via the Web; the remainder (12.8%) completed it via telephone interview. All results presented here are based on the 8619 participants who responded to the question on the intent to be vaccinated.

The majority of the participants were females (69.4%), approximately half (48.2%) were 18–35 years of age, the participants nationalities were Jordanians (43.3%), Palestinians (40.8%) and Syrians (15.9%), more than half of the participants were holding a Bachelor’s degree (51.7%), not working in a health related field (77.6%), employed (61.1%), married (60.1%), and 80% have a family size of ≥4. Participant characteristics are shown in Table 1.

**Table 1:** Demographic characteristics of participants (n = 8619).

Characteristic	Frequencies* n (%)
**Gender**	
Female	5984 (69.4)
Male	2635 (30.6)
**Age group (years)**	
18–35	4155 (48.2)
36–55	3316 (38.5)
56–70	1052 (12.2)
>70	96 (1.1)
**Nationality**	
Jordanian	3735 (43.3)
Palestinian	3516 (40.8)
Syrian	1368 (15.9)
**Education**	
High School diploma or less	1556 (18.1)
Community college	788 (9.1)
Bachelor	4455 (51.7
Graduate degree	1820 (21.1)
**Do you work in any health related field?**	
Yes	1932 (22.4
No	6687 (77.6)
**Employment status**	
Employed	5264 (61.1)
Not employed	3355 (38.9)
**Marital status**	
Married	5180 (60.1)
Widowed	132 (1.5)
Divorced or separated	240 (2.8)
Never been married	3067 (35.6)
**Family size**	
1	144 (1.7)
2	520 (6.0)
3	1064 (12.3)
≥4	6891 (80)

*Data presented as the actual number (percentage) of survey respondents. Percentages may not total to 100 due to rounding.

Most participants (70.6%) perceived that the best way to fight COVID-19 is by wearing a mask and washing hands, 60.6% by social distancing, and 45.8% by vaccination. Additional options for this question and their frequencies are listed in Table 2. Only 32.2% of the participants intend to get vaccinated against COVID-19, while the highest percentage of participants (41.6%) doesn’t intend to get vaccinated or not sure (26.2%). The majority of participants refused or were hesitant to get the vaccine due to concerns about the lack of rigorous evaluation by the FDA (49.1% and 67.3% respectively) or will wait till more many other people try it first (the wait-and-see approach) (33.1% and 66.9% respectively) (Table 2). The frequencies of participants intending to get vaccinated within each characteristic were females (35.9%), 18–35 years old (65.1%), Jordanians (41.3%), holding a Bachelors’ degree (55.4%), employed (54.7%), didn’t have the flu vaccine last year (78.4%), and others (additional frequencies are listed in Table 3).

**Table 2:** Participants views on COVID-19 containment or elimination practices, with the focus on vaccination as one of the options.

Best practices to contain or eliminate COVID-19	Frequencies* n (%)		
	Yes	No	Not sure
**What would be the best way to fight the spreading of the coronavirus?**			
Get vaccinated	3947 (45.8)	4672 (54.2)	
Washing hands and wearing masks	6083 (70.6)	2536 (29.4)	
Herd immunity	1707 (19.8)	6912 (80.2)	
Social distancing	5223 (60.6)	3396 (39.4)	
Avoid contact with sick people	3788 (43.9)	4831 (56.1)	
Using antibiotics	672 (7.8)	7947 (92.2)	
Herbal medicine	432 (5.0)	8187 (95.0)	
Healthy diet	3995 (46.4)	4624 (53.6)	
**Will you take the vaccine if it’s available?**	2772 (32.2)	3589 (41.6)	2258 (26.2)
**Reasons for “no” or “not sure” of getting vaccinated** **			
Not believing in vaccines	655 (7.6)		
Vaccine wasn’t rigorously evaluated by FDA	3055 (35.6)		
Will wait till enough people try it	2220 (25.8)		
COVID-19 is not dangerous	1309 (15.2)		
Getting side effects with any vaccine	328 (3.8)		
Other reasons	1498 (17.4)		

*Frequencies presented as the actual number (percentage) of survey respondents. Percentages may not total to 100 due to rounding.

** * Percentages do not total to 100 because a response could be coded in more than 1 category.

**Table 3:** Intend to be vaccinated by participant demographic characteristics.

Characteristic	Intent to be vaccinated, n (%)*			*P* Value	
	Yes (2772)	No (3589)	Not sure (2258)	Yes vs. No	Yes vs. Not sure
**Gender**				0.002	0.035
Female	2151 (46.3)	2246 (15.9)	1587 (37.7)		
Male	621 (23.6)	1343 (51.0)	671 (25.5)		
**Age group (years)**				<0.001	<0.001
18–35	1805 (65.1)	1466 (40.8)	884 (39.1)		
36–55	698 (25.2)	1559 (43.8)	1059 (39.1)		
56–70	240 (8.7)	505 (14.1)	307 (13.6)		
>70	29 (1.0)	59 (1.6)	8 (0.4)		
**Nationality**				0.003	0.024
Jordanian	1146 (41.3)	1584 (44.1)	1005 (44.5)		
Palestinian	1025 (37.0)	1585 (44.2)	906 (40.1)		
Syrian	601 (21.7)	420 (11.7)	347 (15.4)		
**Education**				<0.001	<0.001
High School diploma or less	832 (30.0)	352 (9.8)	372 (16.5)		
Community college	312 (11.3)	268 (7.5)	208 (9.2)		
Bachelor	1537 (55.4)	1845 (51.4)	1073 (47.5)		
Graduate degree	91 (3.3)	1124 (31.3)	605 (26.8)		
**Do you work in any health related field?**				<0.001	0.004
Yes	655 (23.6)	810 (22.6)	467 (20.7)		
No	2117 (76.4)	2779 (77.4)	1791 (79.3)		
**Employment status**				0.002	<0.001
Employed	1515 (54.7)	2355 (65.6)	1394 (61.7)		
Not employed	1257 (45.3)	1234 (34.4)	864 (38.3)		
**Marital status**				0.047	0.064
Married	1328 (47.9)	2389 (66.6)	1463 (64.8)		
Widowed	53 (1.9)	48 (1.3)	31 (1.4)		
Divorced or separated	80 (2.9)	110 (3.1)	50 (2.2)		
Never been married	1311 (47.3)	1042 (29.0)	714 (31.6)		
**Family size**				<0.001	0.043
1	16 (0.6)	68 (1.9)	60 (2.7)		
2	166 (6.0)	195 (5.4)	159 (7.0)		
3	447 (16.1)	414 (11.5)	203 (9.0)		
≥4	2143 (77.3)	2912 (81.8)	1836 (81.3)		
**Flu vaccine**				0.004	0.024
Participants who had flu vaccine last year	599 (21.6)	398 (11.1)	355 (15.7)		
Participants who didn’t have flu vaccine last year	2173 (78.4)	3191 (88.9)	19.3 (84.4)		
Participants who will be having flu vaccine this year	1232 (44.4)	968 (27.0)	572 (25.3)		
Participants who will not be taking flu vaccine this year	914 (33.0)	2268 (63.2)	1069 (47.3)		
Participants who are not sure if they’ll be taking flu vaccine this year	626 (22.6)	353 (9.8)	617 (27.3)		

*Percentages were calculated based on the answer provided by participants within each characteristic, percentages may not add to 100 due to rounding.

Table 4 summarizes the results for the 27 regressions: one set of nine univariate regressions for the outcomes of the question regarding the intent to get vaccinated against the demographic variables and if the participant had a flu vaccine last year.

**Table 4:** Univariate regression outputs for vaccine acceptability questions against demographic characteristics.

Characteristic	RRR (95% CI) Yes vs No	RRR (95% CI) Yes vs Not sure
**Gender**		
Female vs male	2.1 (1.9, 2.3)	1.5 (1.3, 1.7)
**Age group (years)**		
18–35 vs 36–55	7.5 (6.5, 8.5)	3.1 (2.7, 3.5)
18–35 vs 56–70	0.4 (0.37, 0.41)	0.8 (0.7, 1.0)
18–35 vs >70	0.39 (0.37, 0.41)	0.2 (0.1, 0.5)
**Nationality**		
Jordanian vs Palestinian	1.5 (1.4, 1.7)	1.0 (0.9, 1.1)
Jordanian vs Syrian	0.7 (0.6, 0.8)	0.7 (0.6, 0.8)
**Education**		
High School diploma or less vs Community college	0.72 (0.6, 0.9)	1.1 (1.0, 1.2)
High School diploma or less vs Bachelor	2.3 (2.0, 2.6)	1.1 (0.9, 1.3)
High School diploma or less vs graduate degree	10.3 (8.2, 12.9)	9.5 (7.5, 12.0)
**Do you work in any health related field?**		
Don’t work vs do work in a health related field	0.94 (0.8, 1.0)	0.8 (0.7, 1.0)
**Employment status**		
Employed vs not employed	0.63 (0.6, 0.7)	0.8 (0.7, 0.8)
**Marital status**		
Married vs total of not married	0.27 (0.2, 0.3)	0.1 (0.1, 0.1)
**Family size**		
1 vs 2	0.27 (0.2, 0.5)	0.3 (0.1, 0.5)
1 vs 3	0.8 (0.6, 1.0)	0.5 (0.4, 0.6)
1 vs ≥4	1.5 (1.3, 1.7)	1.9 (1.6, 2.3)
**Flu vaccine**		
Participants who had flu vaccine last year vs who didn’t	1.3 (1.1, 1.4)	1.5 (1.3, 1.8)

People aged 36–55, 56–70 and >70 were less likely to get the vaccine than those who aged 18–35. This difference was the strongest (the relative risk ratio (RRR) = 7.5; 95% confidence interval (CI) (6.5, 8.5) when responses from the middle age cohort and those from the youngest age cohort were compared (Table 4). The same trend was observed with gender, females were more likely to accept the vaccine with RRR of 2.1 (95% CI (1.9, 2.3)). Jordanians were much more likely to get vaccinated (RRR of 1.5 (95% CI (1.4, 1.7)) than Palestinians but less than Syrians (RRR of 0.7 (95% CI (0.6, 0.8)). Higher levels of education were strongly associated negatively with vaccine acceptance, participants a high school diploma or less were ten-fold more intended to be vaccinated than participants with graduate degrees (RRR of 10.3 (95% CI (8.2, 12.9)). Increase in family size of participants reflected positively on the level of acceptance to the vaccine, family size of ≥4 had RRR of 1.5 (95% CI (1.3, 1.7)), the same trend was observed with participants who had the flu vaccine last year (RRR of 1.3 (95% CI (1.1, 1.4)).

## Discussion

The current research aimed at assessing the intent to be vaccinated against COVID-19 among a representative sample of 8619 adults among Jordanians, Palestinians, and Syrians, residing in three countries with high COVID-19 burdens in the Middle East. It also aimed at identifying the predictors of intent to decline or hesitation to get vaccinated against the disease. About one third of the participants (32.2%) intended to be vaccinated, 41.6% declined, and 26.2% were hesitant (Table 2), this is especially striking considering that the survey was conducted during December 2020, when the number of cases and deaths per day due to COVID-19 were at or near peak levels of the initial surge in the three regions. The far-from-willingness to accept the vaccine among the three nationalities is very concerning. Furthermore, reporting one’s willingness, rejection or hesitancy to get vaccinated might not be necessarily a predictor, as vaccine decisions are multifactorial and can change over time. Groups most at risk of mistrust in vaccines are the same groups who also are at increased risk for illness and death from COVID-19; older ages. While Predictors to the intent to be vaccinated were: young ages (18–35) with about 7.5-fold more of getting vaccinated than 36–55 years old, females, Syrians and Jordanians, a family size of ≥4, and having received a flu vaccine last year (Table 3). The surprising fact was the refusal of getting vaccinated increased with the level of education; participants with a high school diploma or less were more than 10-fold willing to get vaccinated than graduate degree holders (Master’s and PhD) (RRR of 10.3 95% CI (8.2, 12.9)), indicating that the refusal of vaccine was not arbitrary but most likely based on specific concerns among educated participants. The reasons for vaccine rejection and hesitancy included the lack of rigorous evaluation of the vaccine by the FDA and hesitancy till many other people try it first (wait-and-see approach), consistent with many other drug studies around the world under normal health situation.

Refusal and hesitancy to vaccination is a common global concern, also it is one of the top ten threats to global health in 2019 according to the World Health Organization [8].

The lack of scientific knowledge and the mistrust in the pharmaceutical industry are thought to be important predictors for vaccine hesitancy. This research was performed in third world countries where immunization is a requirement for school entry as in many countries [9]. Vaccine refusal could have secular and geographical trends, for example, in the USA when nonmedical vaccine exemptions were allowed, the mean exemption rate was doubled [10] this resulted in increasing the individual risk for contagious diseases for instance children that were exempted from vaccinations were 35 times at higher risk to develop measles [11]

In the last months, a huge anti-vaccine content was shared on the social media [9,10] additionally many social media bloggers in the Arab World shared their concerns about the COVID-19 vaccine. Facebook is becoming the population reliable and easy source of medical information with more than 2.2 billion active monthly users [12].

Health Care Providers have an essential influential role in vaccine persuasion. In two studies, individuals were twice as likely to consider vaccines safe after their health care provider counseling [13,14].

The population concerns regarding a potential COVID-19 vaccine efficacy and safety should be the content of interventional educational programs done by the authorities to enhance vaccination rates [15].

Therefore there is an urgent need to familiarize health care providers with the different social media platforms to enhance communication with the population [16]. Recent evidence supports the use of balanced content with a suitable and interactive approach while avoiding complex scientific information [17].

Strengths of our study are that the large, nationally representative sample allows generalization of our findings in each of the three countries under study, and the timing of the survey administration coincided with a peak time of the pandemic in three regions, making the findings particularly timely and prominent.

Our study also has limitations. First, we questioned individuals about their intent to be vaccinated before the vaccination was yet available. It is possible that if more information regarding a potential vaccine was known, some participants might have changed their responses. Second, our study lacked the question(s) to determine what additional information the participant needed about the vaccine to form a solid opinion regarding the acceptance of the potential vaccine.

Although our survey was conducted between the 1^st^ and the 25^th^ of December, 2020, yet the numbers of fully vaccinated percentages among the three countries, as reported between the 13^th^ till the 28^th^ of Sep, 2021, were only 31.88% of the total population in Jordan, 14.52% in the West Bank, and 1.66% in Syria [18]. Future research is required to better access the types of assurances the public needs to be vaccinated, especially when the numbers of COVID-19 cases in three countries are still high.

## References

[1] https://www.who.int/publications/m/item/weekly-operational-update-on-covid-19—26-january-2021.

[2] Nicola M, Alsafi Z, Sohrabi C, Kerwan A, Al-Jabir A, Iosifidis C, et al. The socio-economic implications of the coronavirus pandemic (COVID-19): a review. Int J Surg. 2020;78:185–93. doi: 10.1016/j.ijsu.2020.04.018 ] 32305533 PMC7162753

[3] Phua J, Weng L, Ling L, Egi M, Lim CM, Divatia JV, et al; Asian Critical Care Clinical Trials Group. Intensive care management of coronavirus disease 2019 (COVID-19): challenges and recommendations. Lancet Respir Med. 2020;8:506–17. doi: 10.1016/S2213-2600(20)30161-2 32272080 PMC7198848

[4] Randolph HE, Barreiro LB. Herd immunity: Understanding COVID-19. Immunity 2020;52(5):737–41 May 19. doi: 10.1016/j.immuni.2020.04.012 32433946 PMC7236739

[5] Altmann DM, Douek DC, Boyton RJ. What policy makers need to know about COVID-19 protective immunity. The Lancet 2020;395(10236):1527–9 May 16. doi: 10.1016/S0140-6736(20)30985-5 32353328 PMC7185915

[6] de Figueiredo A, Simas C, Karafillakis E, Paterson P, Larson HJ. Mapping global trends in vaccine confidence and investigating barriers to vaccine uptake: A large-scale retrospective temporal modelling study. The Lancet 2020 [Internet] Sep 10 [cited 2020 Sep 23]; Available from: http://www.sciencedirect.com/science/article/pii/S0140673620315580. doi: 10.1016/S0140-6736(20)31558-0 32919524 PMC7607345

[7] https://www.irishtimes.com/news/world/uk/thousands-of-covid-19-sceptics-and-anti-vaxxers-protest-in-london-1.4342128

[8] Thunstrom L, Ashworth M, Finnoff D, Newbold S. Hesitancy towards a COVID-19 vaccine and prospects for herd immunity. Available at SSRN 3593098. 2020. 10.1007/s10393-021-01524-0 PMC8175934 34086129

[9] Gunaratne K, Coomes EA, Haghbayan H. Temporal trends in anti-vaccine discourse on twitter. Vaccine. 2019;37(35):4867–71. doi: 10.1016/j.vaccine.2019.06.086 31300292

[10] Omer SB, Pan WK, Halsey NA, Stokley S, Moulton LH, Navar AM, et al. Nonmedical exemptions to school immunization requirements: secular trends and association of state policies with pertussis incidence. JAMA 2006; 296:1757–63. doi: 10.1001/jama.296.14.1757 17032989

[11] Salmon DA, Haber M, Gangarosa EJ, Phillips L, Smith NJ, Chen RT. Health consequences of religious and philosophical exemptions from immunization laws: individual and societal risk of measles. JAMA 1999;282:47–53. [Erratum, JAMA 2000;283:2241.] doi: 10.1001/jama.282.1.47 10404911

[12] Betsch C, Renkewitz F, Betsch T, Ulshöfer C. The influence of vaccine-critical websites on perceiving vaccination risks. J Health Psychol. 2010; 15(3):446–55. doi: 10.1177/1359105309353647 20348365

[13] Smith PJ, Kennedy AM, Wooten K, Gust DA, Pickering LK. Association between health care providers’ influence on parents who have concerns about vaccine safety and vaccination coverage. Pediatrics 2006; 118(5):e1287–e1292. doi: 10.1542/peds.2006-0923 17079529

[14] Robichaud P, Hawken S, Beard L, Morra D, Tomlinson G, Wilson K, Keelan J. Vaccine-critical videos on YouTube and their impact on medical students’ attitudes about seasonal influenza immunization: A pre and post study. Vaccine. 2012; 30 (25):3763–70. doi: 10.1016/j.vaccine.2012.03.074 22484293

[15] Harrison EA, Wu JW. Vaccine confidence in the time of COVID-19. Eur J Epidemiol. 2020; 35:325–30.) doi: 10.1007/s10654-020-00634-3 32318915 PMC7174145

[16] Yammine S. Going viral: how to boost the spread of coronavirus science on social media. Nature. 2020;581(7808):345–46. doi: 10.1038/d41586-020-01356-y 32371962

[17] Shoup JA, Wagner NM, Kraus CR, Narwaney KJ, Goddard KS, Glanz JM. Development of an interactive social media tool for parents with concerns about vaccines. Health Educ Behav. 2015;42(3):302–12. doi: 10.1177/1090198114557129 25413375 PMC7651778

[18] https://covidvax.live/, last accessed on Sep, 30th, 2021.

